# Assembly and Connection of Micropatterned Single Neurons for Neuronal Network Formation

**DOI:** 10.3390/mi9050235

**Published:** 2018-05-15

**Authors:** Shotaro Yoshida, Midori Kato-Negishi, Shoji Takeuchi

**Affiliations:** 1 Center for International Research on Integrative Biomedical Systems, Institute of Industrial Science, The University of Tokyo, 4-6-1 Meguro-ku, Komaba, Tokyo 153-8505, Japan; syoshida@iis.u-tokyo.ac.jp (S.Y.); negishi@iis.u-tokyo.ac.jp (M.K.-N.); 2 International Research Center for Neurointelligence (WPI-IRCN), The University of Tokyo Institutes for Advanced Study (UTIAS), The University of Tokyo, Tokyo 153-8505, Japan

**Keywords:** neuronal network, micropatterning, micromanipulation, hippocampal neuron, synapse

## Abstract

Engineering of neuronal network geometry by micropatterning technology is a key future technology for creating artificial brains on a chip. However, engineering of network geometry at the single-cell-level with functional morphology (axon/dendrite) and connectivity (synapses) is still challenging. Here, we describe a method for controlling the axon and dendrite morphology of single primary-cultured neurons and assembling a neural circuit using mobile microplates. The microplates enabled morphological control of neurons by their shapes and bringing their ends into contact caused the formation of physical connections. Functional synapse formation at the connection was indicated by immunostaining of synapse-related proteins and intracellular Ca^2+^ imaging of neural activity. We believe that the method will be useful in engineering neural circuits with selected neurons and defined morphology.

## 1. Introduction

The brain, the sophisticated biological information processor in a living organism, is fundamentally constructed as a neuronal network, an assembly of a hundred billion cell bodies connected by elongated axons and dendrites [1]. Anatomical and functional investigation of this complex neuronal network is extremely difficult in vivo [2]. In vitro models of the network serve as critical research tools due to their simplicity of network structure and the controllability of their physical and chemical environments [3].

Micropatterning technologies including lithography, molding, laser structuring are recently developed tools for preparation and control of in vitro neuronal networks [4,5,6,7,8,9,10,11]. Micropatterning technologies enable manipulation of physical and chemical guidance cues for cultured neurons, e.g., cell-sized barriers or trenches for guiding axon and dendrite growth [12], and micropatterned protein-based islands for guiding the location of cell bodies [13]. With these tools a researcher can design a set of guidance cues and thereby determine the geometry of the neuronal network [14]. Researchers have found three important neuronal behaviors relating to micropatterns: (i) When a neuron is seeded on a cell-adhesive circular micropattern with several protruding lines, the cell body of the neuron tends to adhere to the circle and its neuronal processes (axon and dendrites) tend to grow along the lines [15,16]; (ii) When neurons are seeded on an array of circles connected by lines, the positions of the cell bodies and neuronal processes (that is, the geometry of the neuronal network) are roughly determined by the circles and lines respectively [17,18]; (iii) The lengths of linear micropatterns influence differentiation of axons and dendrites from immature neuronal processes [19,20]. By utilizing the behavior of neurons on circle and line micropatterns, it is possible to design a neuronal network geometry, and thereby to construct an artificially designed brain on a chip. A significant drawback of the technique is that the success rate of positioning and connection are not 100% (less than 10% in practice), due to the variability of growth and the immobility of neurons in culture [21]. As a consequence, full control over neuronal network geometry at the single-cell level is virtually impossible. One possible approach to solving the problem is, as we previously reported [22], to mobilize micropatterned single neurons by attaching them to movable microplates. Recently we have demonstrated that a circular- or linear-shaped microplate can control the positions of the cell bodies and neurites of single neuron-like cells (PC12 cells), and their positions can be changed on demand during culture by micro-manipulating the microplates. This technique allows for the positioning of cell bodies and neurites to desired locations with 100% certainty, and thus forms a promising approach to the creation of fully controlled neuronal networks at the single-cell level. However, since the PC12 neuron-like cells did not have specification of axon and dendrites, and did not have synaptic connection, it was unclear whether the microplate technique can be used to form functional neuronal networks.

**Figure 1:**
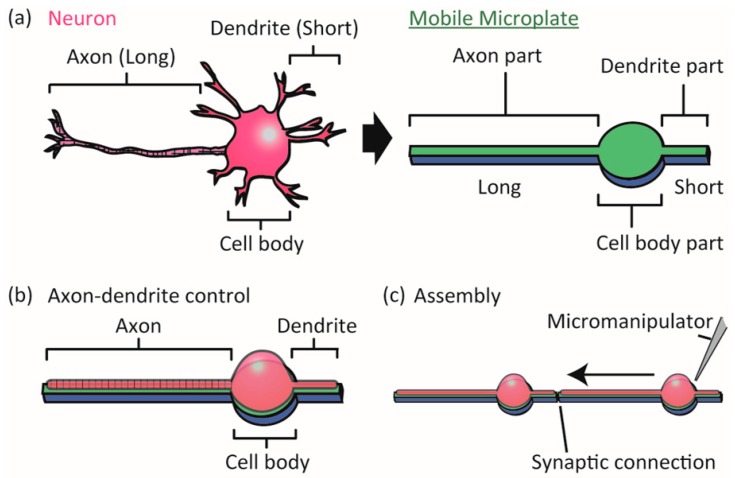
Concept of the study. (**a**) We designed a device, which we call a mobile microplate, as a micro-sized flat plate with three parts for positioning the axon, dendrite, and cell body of a neuron; (**b**) The microplate enabled controlling axon-dendrite morphology as well as the cell body location of a neuron; (**c**) The micropatterned single neurons on the microplates could be joined by a micro-manipulator to form functional synaptic connections. The device is intended to be useful in designing neuronal networks on a chip for biophysical neuroscience studies of topics such as synapse formation and neuronal signal processing.

In this paper, we investigate the next step in the microplate-based neuronal network formation technique, namely control of functional axon and dendrite formation on the microplates and functional synaptic connection between the neurons. Since PC12 cells do not have functional axons, dendrites and synapses, we employ rat primary-cultured hippocampal neurons and newly designed microplates composed of a circle and protruding long and short arms (Figure 1). We expect the cell bodies of hippocampal neurons to attach to the circular areas (Cell body parts), the axons to grow along the long arms (Axon parts), and the dendrites to grow along the short arms (Dendrite parts). The microplates have a biocompatible sacrificial layer underneath for further detachment and manipulation, and are massively arrayed on a non-cell-adhesive background to allow for the stochastic capture of single neurons on the microplates. We first examine whether the long and short arms of microplates can control the differentiation of axons and dendrites. We then assemble the cultured neurons on the microplates using a micro-manipulator, and investigate whether synaptic connections form.

## 2. Materials and Methods

### 2.1. Design of Mobile Microplates

We designed the microplates to have a central Cell body part consisting of a 30 μm-diameter circle, a long Axon part consisting of a 100 μm straight line, and one to three short Dendrite parts consisting of 20 μm lines. The thickness of the microplate was 2 μm. Microplates were arrayed on glass with 100 μm gaps in the *x* and *y* directions. The surface of the glass was covered with cell-repulsive 2-methacryloyloxyethyl-phosphorylcholine (MPC) polymer [23] to pattern neurons only on the microplates. The microplates were composed of three layers: a gelatin sacrificial layer for detachment of the microplate from the glass after cell culture, a 2 μm transparent parylene layer for adhesion and manipulation of neurons, and a laminin cell-adhesive layer for promoting neuronal adhesion. The gelatin was biocompatible and denatured during culture at 37 °C to enable detachment of the parylene layer by pushing with a glass capillary [22]. The parylene layer was biocompatible and enabled phase contrast and fluorescent microscopy of the cells [22]. Laminin was chosen for its affinity to primary hippocampal neurons.

### 2.2. Fabrication of Mobile Microplates

**Figure 2:**
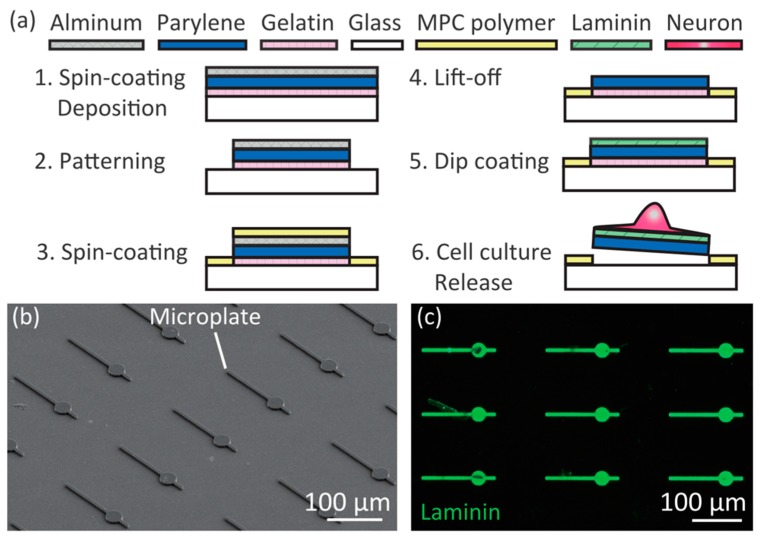
Fabrication of the microplate device. (**a**) Fabrication process; (**b**) Scanning electron microscopy (SEM) image of fabricated microplates on glass; (**c**) Immunostained image of laminin (green) on the microplates.

We fabricated the mobile microplates by a photolithography process using a protein micropatterning technique (Figure 2a). The fabrication process was slightly modified from our previous report [22]. First, we cleaned the surface of 120–170 μm-thick glass by acetone and isopropanol washing, treated it with O_2_ plasma for hydrophilization, and spin-coated 0.01% (*w*/*v*) gelatin (from porcine skin, type A, Sigma-Aldrich, St. Louis, MO, USA) at 2000 rpm for 30 s on the glass. After dehydrating in a vacuum chamber for more than 2 h, 2–3 µm thick parylene C (DPX-C, Speedline Technology, Franklin, MA, USA) and aluminum (Al) were deposited. A photoresist layer (S1818, Shipley, Marlborough, MA, USA) was patterned to the microplate shapes using photolithography, then the exposed Al layer was chemically etched by 2.38% tetramethyl ammonium hydroxide solution (NMD-3, Tokyo Ohka Kogyo Co., Ltd., Tokyo, Japan). Exposed Parylene C and gelatin layers were dry-etched by O_2_ plasma. MPC solutions (provided by Prof. K. Ishihara of the University of Tokyo) were spin-coated at 2000 rpm for 30 s followed by drying under an ethanol atmosphere for 20 min, and baked at 70 °C for 4 h. Before cell culture, the Al and MPC polymers were removed from the microplates by NMD-3, and the device was placed in a 35 mm-diameter glass dish. Laminin (L2020, Sigma, St. Louis, MO, USA) was dissolved for 2 μg/mL concentration in a basal salt solution composed of 130 mM NaCl (Wako, Osaka, Japan), 5.4 mM KCl (Wako), 1.8 mM CaCl_2_ (Kanto Chemical, Tokyo, Japan), 5.5 mM d-glucose (Kanto Chemical), 20 mM 4-(2-hydroxyethyl)-1-piperazineethanesulfonic acid (HEPES, Wako) of pH 7.4, and coated to the surface of the device for 100 μL/cm^2^, incubated for 4 h in a humidified 37 °C incubator, and washed with phosphate buffer saline without MgCl_2_ and CaCl_2_ (PBS, Sigma) four times. Then 2 mL of Neurobasal medium (Sigma) supplemented with 2% B27 (Gibco, Waltham, MA, USA), 1% GlutaMAX (Gibco), 1% penicillin-streptomycin (Sigma-Aldrich) was poured in the dish, and incubated in a humidified 37 °C incubator.

We confirmed that the fabricated microplates were as designed by SEM observation (Figure 2b). To investigate whether laminin layers were formed only on the microplates, we immunostained for laminin using rabbit polyclonal anti-laminin primary antibody (AB11575, Abcam, Cambridge, UK) and secondary antibody anti-rabbit IgG-AlexaFluor488 (A-11008, Invitrogen, Waltham, MA, USA) (Figure 2c). Laminin was detected only on the microplates. We concluded that the micropatterning of the laminin was a result of the anti-protein absorption function of the MPC polymers on the glass.

### 2.3. Cell Culture

E16-20 rat primary hippocampal cells were obtained by a standard protocol [24]. All rats were treated in accordance with the policies of the University of Tokyo Institutional Animal Care and Use Committee. Primary hippocampal cells were seeded on the microplates at 1.5 × 10^2^ cells/mm^2^ and cultured in Neurobasal medium supplemented with 2% B27, 1% GlutaMAX, 1% penicillin-streptomycin. The medium was changed after 1 day to remove unattached cells. The medium was routinely replaced once a week.

### 2.4. Gene Manipulation

Primary hippocampal neurons on microplates were genetically manipulated by adeno-associated virus vectors to express green fluorescent protein (GFP, AV-9-PV1917, Penn Vector Core) or GCaMP6 (intracellular Ca^2+^ indicator, AV-1-PV2822, Penn Vector Core) under the control of a neuron-specific promoter. A solution of virus vectors was added to the culture medium, and the infected neurons on the microplates were observed after 1 day.

### 2.5. Immunostaining of Neurons on Microplates

Neurons were fixed by 4% paraformaldehyde (Wako) for 1 h, permeabilized with 0.5% TritonX100 (Alfa Aesar, Lancashire, UK) for 6 min, and incubated in 1% bovine serum albumin (BSA, Sigma) for 1 h. The fixed cells were treated with mouse monoclonal anti-Tau-1 (axon marker, 1:1000, MAB3420, Millipore, Burlington, MA, USA), rabbit polyclonal anti-microtubule-associated protein 2 (MAP2, dendrite marker, 1:1000, AB1543, Millipore), guinea pig polyclonal anti-vesicular glutamate transporter 1 (vGlut-1, pre-synapse marker, 1:1000, AB5905, Millipore), or mouse monoclonal anti-PSD95 (post-synapse marker, 1:1000, ab2723, Abcam) primary antibody at 4 °C overnight. The neurons were then reacted with anti-mouse IgG-AlexaFluor488 (A11001, Invitrogen) or anti-rabbit IgG-AlexaFluor568 (A11011, Invitrogen) or anti-rabbit IgG-AlexaFluor647 (A-21245, Invitrogen) or anti-Guinea Pig IgG-AlexaFluor488 (A-11073, Invitrogen) for 1 h at room temperature.

### 2.6. SEM Imaging

We sputtered platinum (Pt) on the microplates before scanning electron microscope (SEM, SU-8000 FESEM, Hitachi High-Technologies Co., Tokyo, Japan) observation. Neurons on the microplates were freeze-dried before SEM observation. We fixed the neurons by replacing the culture medium with 2% glutaraldehyde (Wako) solution and waited for 15 min. The microplates were then washed with pure water three times, and washed with 30%, 50%, 70% ethanol for one time each. After 10 min, the microplates were immersed in 80%, 90%, 95%, 99.5% ethanol for 10 min each and washed with 99.5% ethanol two times. We immersed the microplates in a tert-butyl alcohol solution (Wako) for 10 min and repeated this a total of three times. The culture dish with microplates was covered by parafilm, and frozen in a −80 °C freezer for 5 min. Finally, the microplates were dried in a freeze-drier (FS-2030, Hitachi, Tokyo, Japan), sputtered with Pt, and observed by SEM. 

### 2.7. Image Acquisition and Processing

Inverted microscopes (IX71 or IX81, Olympus, Tokyo, Japan) were used to take phase contrast and fluorescent images. In Ca^2+^ imaging, culture medium was replaced with basal salt solution (described in Section 2.2) to induce spontaneous neuronal activity, then imaged by real-time confocal microscopy (LSM710, Zeiss, Oberkochen, Germany). Time-lapse movies were taken with inverted microscopes (Olympus IX81) equipped with a humidified 37 °C incubator chamber containing 5% CO_2_. The images were processed using ImageJ (NIH) to enhance their contrast for visualization. The stained cells were pseudo-colored by ImageJ for visibility.

### 2.8. Handling of Single Neurons on Microplates

The tip of a glass capillary was heated and pulled to attain a diameter of 1 μm, then coated with 1 μm parylene. The parylene-coated glass capillary was attached to a micro-manipulator. We handled single neurons on microplates by pushing the side of a microplate with the parylene-coated glass capillary. The manipulation procedures were conducted in less than 15 min to avoid large pH and temperature changes.

## 3. Results

### 3.1. Controllability of Position of Cell Body/Axon/Dendrites on Microplates

**Figure 3:**
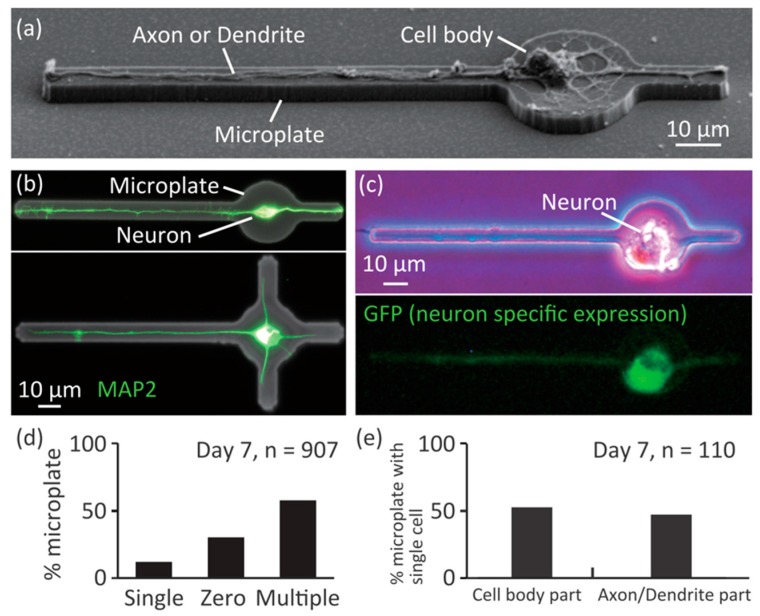
Culturing single neurons on the microplates. (**a**) A SEM image of a single neuron on a microplate; (**b**) Immunostained neurons on microplates; green: MAP2; (**c**) GFP expression under control of excitatory-neuron-specific CaMKII promoter; (**d**) Histogram of number of cells on individual microplates; (**e**) Histogram of positions of cell body of single neurons on microplates.

To investigate whether single primary hippocampal neurons were patterned on microplates, we seeded neurons and observed the results by SEM and fluorescent microscopy (Figure 3). At 6 days after seeding onto microplates, neurons were observed by SEM (Figure 3a). The cell bodies were positioned on the cell body parts, and neuronal processes (axons or dendrites) grew only on the Axon and Dendrite parts. We observed stained neurons on the microplates by MAP2 immunostaining (Figure 2b), and found that the neuronal processes could be patterned even when the number of Dendrite parts was three. We previously found that the success rate of patterning neuronal processes of PC12 neuron-like cells decreased as the number of lines increased [22], thus we used microplates with one Dendrite part for the rest of the experiments to make the success rate as high as possible. To check whether the cell on a microplate was a neuron or another type of brain cell, such as a glial cell, we specifically marked neurons on the microplates by expressing GFP under a neuron-specific promoter by transfection with an adeno-associated virus (Figure 3c). The cells on the microplates showed green fluorescence, confirming that they were indeed neurons.

To quantify the success rate of single neuron patterning on the microplates, we counted the number of cells on the microplates at day 7 after cell seeding (Figure 3d). The success rate of single cell positioning was approximately 10%, and multiple cells tended to attach on the microplates. The low success rate might be improved in a future study by employing precise cell positioning techniques such as microfluidic devices. We checked the position of cell body on the successful samples (Figure 3e), and found approximately half of the cell bodies were attached on the cell body part, but the rest were attached to the Axon or Dendrite parts. In our previous study, PC12 neuron-like cells migrated from the line part to circle part, however, we observed that the cultured neurons did not migrate very frequently. The low rate of accurate positioning of the cell body might also be improved by precise cell positioning techniques in the future.

**Figure 4:**
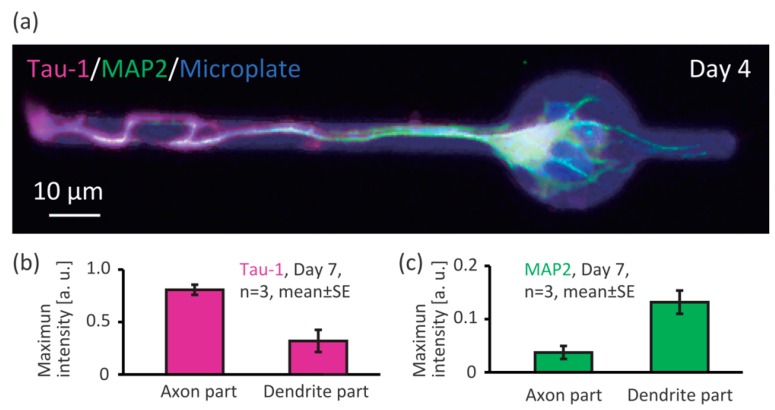
Axon-dendrite control of single neurons by the shape of the microplates. (**a**) Immunofluorescent image of a neuron on a microplate (Tau-1: axon marker, MAP2: dendrite marker); Maximum intensity of (**b**) Tau-1; and (**c**) MAP2 on the Axon and Dendrite parts of the microplate.

To investigate whether the Axon and Dendrite part of the microplates enable patterning of the axon and dendrites of single neurons, we immunostained for Tau-1 (axon marker) and MAP2 (dendrite marker) and observed the results by fluorescent microscopy (Figure 4). At day 4 after cell seeding, we found a neuron on a microplate whose axon was on the Axon part and dendrite on the Dendrite part (Figure 4a, Tau-1: magenta, and MAP2: green, pseudo-colored). We measured intensity of the Tau-1 and MAP2 marker fluorescence, and found that the Tau-1 signal (axon) were higher on the Axon part (Figure 4b), and the MAP2 signal (dendrite) were higher on the Dendrite part (Figure 4c). Therefore, we can say that the shape of the microplate enabled control of axon-dendrite morphology. 

### 3.2. Assembly and Connection of Neurons on the Microplates

**Figure 5:**
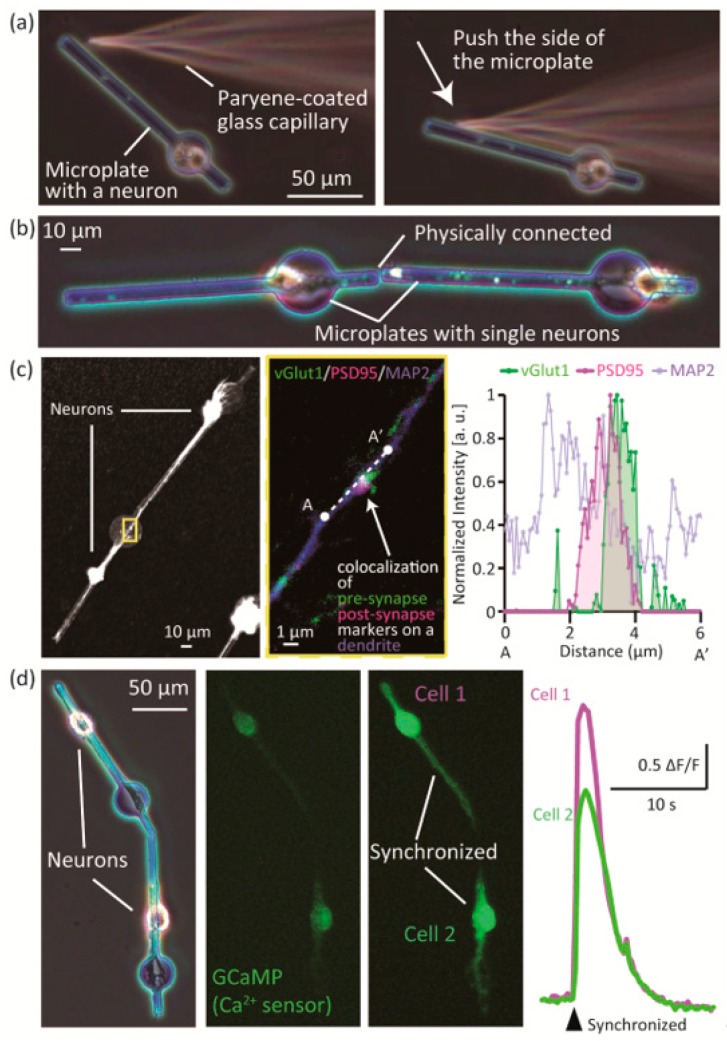
Assembly of single neurons on mobile microplates. (**a**) Micromanipulation of a microplate with a neuron; (**b**) Two adjoining neurons that were physically connected; (**c**) Immunostaining for vGlut1 (pre-synaptic), PSD95 (post-synaptic) and MAP2 (dendritic) on adjoined neurons; (**d**) Ca^2+^ imaging of adjoined neurons.

To confirm that the position of the neuron-laden microplates can be changed, we manipulated the microplates by a glass capillary tube controlled by a micro-manipulator (Figure 5a). After 4 days of culture, the microplate could be manipulated by pushing the side with the tip of a glass capillary tube. Two morphologically controlled single neurons on microplates could both be micromanipulated, and adjoined during culture (Figure 5b, Video S1). To show that the assembled neurons could form functional synaptic connections, we immunostained the assembled neurons for synapse markers (vGlut1: pre-synaptic, PSD95: post-synaptic) and MAP2 (Figure 5c). We observed that pre-synaptic marker vGlut1 and post-synaptic marker PSD95 co-localized at a spot on a dendrite stained by MAP2. We measured the fluorescence intensity of the spot and plotted the normalized intensity (Figure 5c, right graph)**.** The graph shows that the signals of vGlut1 and PSD95 overlap with the MAP2 signal, which suggests that a synaptic connection was formed by the adjoined neurons.

Finally, we observed a synaptic connection of the adjoined neurons by measuring neuronal activity (Figure 5d, Figure S1, Video S2). The neurons were transfected by adeno-virus to express GCaMP6, which emits green fluorescent signals depending on the intracellular Ca^2+^ concentration. An example is shown in the left and center panel of Figure 5d. Two single neurons on microplates were preliminarily assembled by micro-manipulation, and expressed GCaMP6. We traced the time course of fluorescence change of the GCaMP6, and found that the signals of the two neurons synchronized at a moment (Figure 5d, right panel, Figure S1). The graph in Figure 5d shows the changes in fluorescence signals of the cell bodies of the two cells in the left panel (F: initial fluorescent intensity, ΔF: change in fluorescent intensity between neighboring frames of the time lapse movie). We observed that the fluorescence signals of the two neurons—representing neuronal activity—were synchronized. As shown in Figure S1, activities of the two neurons synchronized at a certain moment, while those of other neurons on neighbor microplates did not synchronized. This result indicates that the overlap of traces of the two cells was not coincidental but synapse-related synchronization (a synaptic connection was formed between the two neurons).

## 4. Discussion

The newly designed microplate enabled culturing of single primary hippocampal neurons and control of their axon-dendrite morphology. The morphologically controlled single neurons could be micromanipulated and assembled during culture. We observed synaptic connections between adjoined neurons by immunostaining for co-localized synapse-related proteins, and by measurement of synchronization of neuronal activity with intracellular Ca^2+^ imaging. For the first time, the mobile microplate method enabled changing the position of morphologically controlled single primary neurons, since in a conventional setup they die when they are detached from the culture. The method may be useful in designing neuronal networks with single cell-resolution, which is difficult to achieve by conventional micropatterning techniques.

The success rate of single neuron positioning was as low as in previous studies (less than 10%). There were three possible reasons of the low rate. First, neurons that were randomly seeded on a microplate aggregated, and single cells were rarely found. To overcome the multiple cell aggregation, it will be necessary in the future to precisely position single neurons on each microplate by microfluidic techniques such as droplet-based single cell isolation and positioning [25]. Second, neuronal cell bodies and neuronal processes tended to localize to undesired parts of the microplate. As shown in Figure 5c,d, cell body sometimes migrated toward the Axon and Dendrite parts. This problem was probably a result of the size and structure of the Cell body, Axon, and Dendrite parts of the microplates. It will be necessary to optimize the size of the microplates (e.g., fabricating a narrower Axon and Dendrite parts to prevent intrusion of soma from Cell body part, since soma tends to adhere on wide area) and add structures such as walls on the microplates to avoid having cell bodies migrated to the Axon and Dendrite parts. Finally, single neurons tended to die when they were cultured alone. Co-culturing with glia and connection with other neurons may be important factors for neuronal survival [26], thus preparation and assembly of glia-laden microplates and detailed investigation of the relationship between neuronal cell death and time before assembly are needed.

Although we observed clear examples of functional synaptic connection between assembled neurons by immunostaining and Ca^2+^ imaging, it was difficult to observe assembled neurons with well-controlled morphology, and this hampered quantification of synaptic connections. Regarding immunostaining, it was necessary to refresh the medium around neurons many times during fixing and antibody treatment, which frequently disassembled the assembled microplates, since they could easily be moved by the drag force of the medium. For more precise analysis, it will be necessary to add microfabricated structures to stabilize the assembled microplates. In Ca^2+^ imaging, although it did not require medium exchange, imaging of well-controlled neurons was difficult. Since the assembled microplates were often disassembled when we merely transported the whole culture dish to another place, the microplate device was essentially not able to be moved, and only one point was observable. Combined with the low success rate of morphological control of single neurons on the microplates, this made it very difficult to repeat assembly and Ca^2+^ imaging. To improve the results, the success rate of morphological control of single neurons on a microplate needs to be increased and a microfabricated structure for stabilizing the assembled microplates is needed, as mentioned above.

Synchronization of assembled neurons observed in Figure 5 was considered to be synapse-oriented by the long traces shown in Figure S1. Neuronal activities of the two neurons did not synchronize at 1.7 s, and synchronized at 34.8 s, indicating that the input signal of pre-synaptic cell 2 was not sufficient to fire post-synaptic cell 1, or action potential of the post-synaptic cell 2 did not affect pre-synaptic cell 1. We could not clearly distinguish pre or post synaptic neurons from the results, however, as already described in Section 3.2, the overlap of activities was not coincidental but synapse-related synchronization since activities of the other neurons on neighbor microplates did not synchronized at the same time.

## 5. Conclusions

We developed mobile microplates that enable axon-dendrite morphology control and assembly of single primary neurons. A synaptic connection between assembled neurons was indicated. We believe the method will be useful in engineering neuronal networks at the single-cell-level resolution to develop artificially designed brain on a chip.

## Supplementary Materials

The following are available online at http://www.mdpi.com/2072-666X/9/5/235/s1, Figure S1: Long traces of intracellular Ca^2+^ of adjoined neurons. Video S1: Assembled neurons on microplates, Video S2: Synchronization_x8_speed.wmv.
