# Supplementary material for: Assembly and Connection of Micropatterned Single Neurons for Neuronal Network Formation
Source: Micromachines (Basel). 2018 May 15;9(5):235. doi: 10.3390/mi9050235 (PMC6187671; doi:10.3390/mi9050235)
Supplement: Supplementary file 1 [file micromachines-09-00235-s001.pdf]

Supplementary Materials: Assembly and Connection of Micropatterned Single Neurons for Neuronal Network Formation

Shotaro Yoshida, Midori Kato-Negishi and Shoji Takeuchi

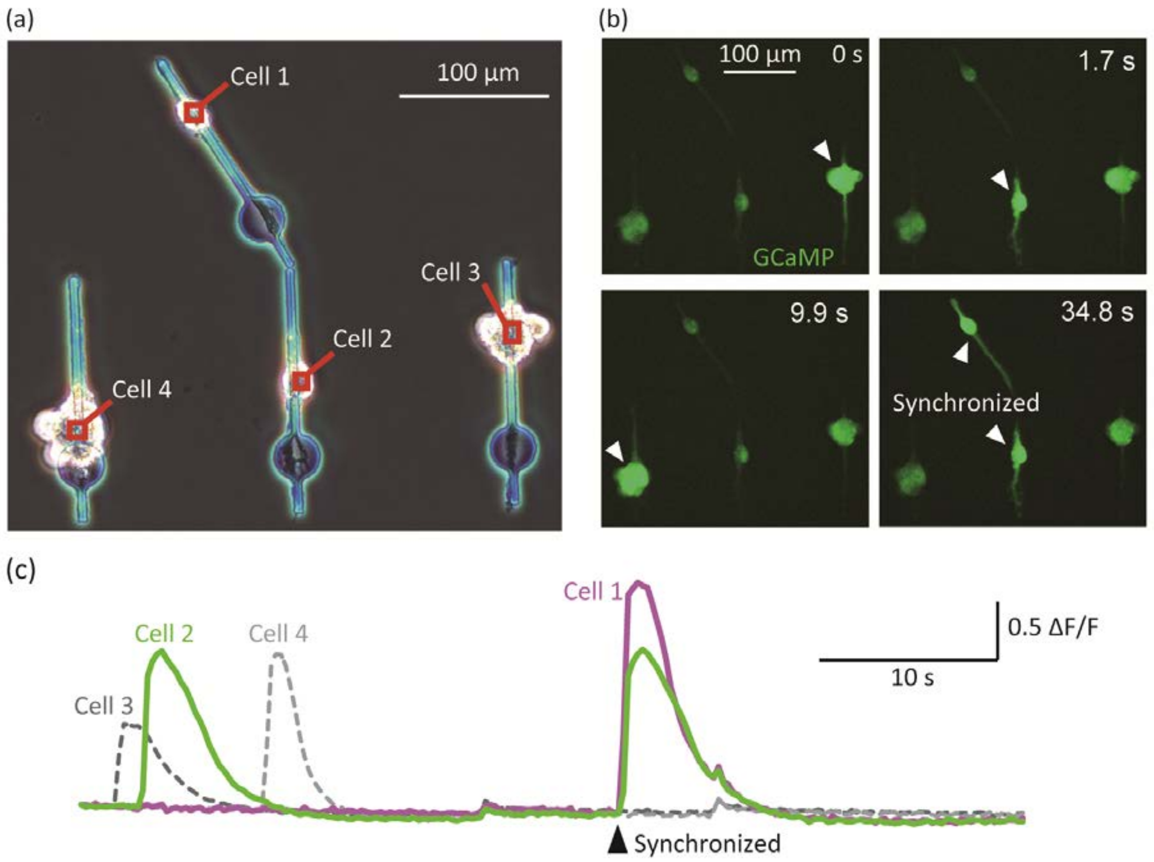

Figure S1. Long traces of intracellular Ca^{2+} of adjoined neurons. (**a**) Four microplates with neurons adhered. The two microplates at the center have single neurons as shown in Figure 5d. (**b**) Fluorescence of GCaMP6 at 0, 1.7, 9.9, 34.8 s. The fluorescence of each neuron increased at each frame. (**c**) Long traces of averaged fluorescent intensities of cell 1, 2, 3, 4 in (a). The cell 1 and 2 synchronized at 34.8 s.

© 2018 by the authors. Submitted for possible open access publication under the terms and conditions of the Creative Commons Attribution (CC BY) license (http://creativecommons.org/licenses/by/4.0/).
